# Overall Results for a National Program of Photodynamic Therapy for Basal Cell Carcinoma: A Multicenter Clinical Study to Bring New Techniques to Social Health Care

**DOI:** 10.1177/1073274819856885

**Published:** 2019-06-26

**Authors:** Hilde Harb Buzzá, Lilian Tan Moriyama, José Dirceu Vollet-Filho, Natalia Mayumi Inada, Ana Paula da Silva, Mirian Denise Stringasci, Michelle Barreto Requena, Cintia Teles de Andrade, Kate C. Blanco, Dora Patricia Ramirez, Cristina Kurachi, Ana Gabriela Salvio, Vanderlei S. Bagnato

**Affiliations:** 1 São Carlos Institute of Physics, University of São Paulo (USP), São Carlos, São Paulo, Brazil; 2 Instituto Federal de Alagoas, Campus Piranhas, Brazil; 3 Skin Department, Amaral Carvalho Foundation, Jaú, Brazil

**Keywords:** photodynamic therapy, PDT, nonmelanoma skin cancer, BCC, basal cell carcinoma

## Abstract

Along the past years, a national program to implement photodynamic therapy (PDT) for nonmelanoma skin cancer (NMSC) was performed over the Brazilian territory. Using a strategy involving companies, national bank, and medical partners, equipment, medication, and protocols were tested in a multicenter study. With results collected over 6 years, we could reach a great deal of advances concerning the use of PDT for skin cancer. We present the overall reached results of the program and discuss several aspects about it, including public politics of treatment. A discussion about advantages of this technique within conditions of health care is placed, comparing PDT with surgery, including an analysis about the implementation of PDT in countries in development as Brazil, considering not only technical but social aspects, as the distribution of medical doctor in the Brazilian territory. The program resulted in a huge dissemination of PDT in Brazil and many countries in Latin America, in a partnership among public politics, universities, companies, and hospitals and clinics and in the insertion of national technologies as option to treat NMSC. Consequence of the program is mainly the continuation of the use of PDT in Brazil and many countries in Latin America.

## Introduction

The worldwide incidence rate of nonmelanoma skin cancer (NMSC) is high compared to other cancer types and is increasing. The incidence of NMSC varies with geographical location according to factors such as exposure to ultraviolet radiation, sun-seeking behavior, and skin type.^1^ In Brazil, the scenario is not different, and according to the Brazilian National Cancer Institute José Alencar Gomes da Silva, the assisting body of the Ministry of Health for the development and coordination of integrated actions for cancer prevention and control in Brazil, 600 000 new cases of cancer per year are estimated for 2018 to 2019 biennium. Nonmelanoma skin cancer will contribute with about 170 000 new cases per year, being the most incident tumor type in Brazil.^2^ The situation goes beyond a health problem, transforming in an economic and logistic concern.

There are 2 main types of NMSC: basal cell carcinoma (BCC) and squamous cell carcinoma (SCC). Rapid growth, increased local invasion, and metastatic potential are some characteristics of SCC.^3^ In contrast, BCC is the most prevalent skin cancer type and is characterized by slow growth and local infiltration.^3^ Although NMSC does have a low mortality rate, it still has a significant impact on quality of life and is placing a large financial burden on health-care services.^1^ In the United States, it was estimated that approximately 3.1 million cases of NMSC were treated from 2002 to 2006, and this number increased up to 4.3 million from 2007 to 2011.^4^ This incidence increase resulted in a raise of the average annual total cost for NMSC care over 170%, while the average annual total cost for all other cancers increased by only 25.1%.^5^

A report published in 2011 on the financial burden to the public and private health-care systems only in São Paulo state, Brazil, revealed that spending on the treatment of NMSC accounted near R$63 million (about US$37 million) annually; added to this, we have a very large expense with the public health system. The diagnosed cases of NMSC in São Paulo state represents about 23% of the new cases in the country,^2^ which means that NMSC may cost near US$200 million annually to the Brazilian health systems.

Although BCC does not present a mortality risk, it usually represents a major impact on the patient’s health and welfare due to the tumor’s ability to invade and destroy adjacent tissue, resulting in multiple lesions and frequent recurrence. Conventional treatments for BCC lesions include chemical and surgical techniques, the latter being the most efficient procedure for the treatment of those lesions.^6^ However, surgery greatly impacts the Brazilian health system as it demands an adequate infrastructure and specialized human resources. As the number of new cases tends to increase, all the required resources (financial, human, infrastructure) will have to increase according. In addition, surgery may result in patient morbidity, especially considering anatomic functionality and unsatisfactory cosmetic results. Surgery is presently the gold-standard therapeutics for this type of cancer, with the curative rate around 96%.^7^ Nevertheless, it is important to mention that most part of the patients are elderly individuals and they are often susceptible to venous and arterial insufficiencies that may compromise the healing process.^8,9^

**Figure 1.**
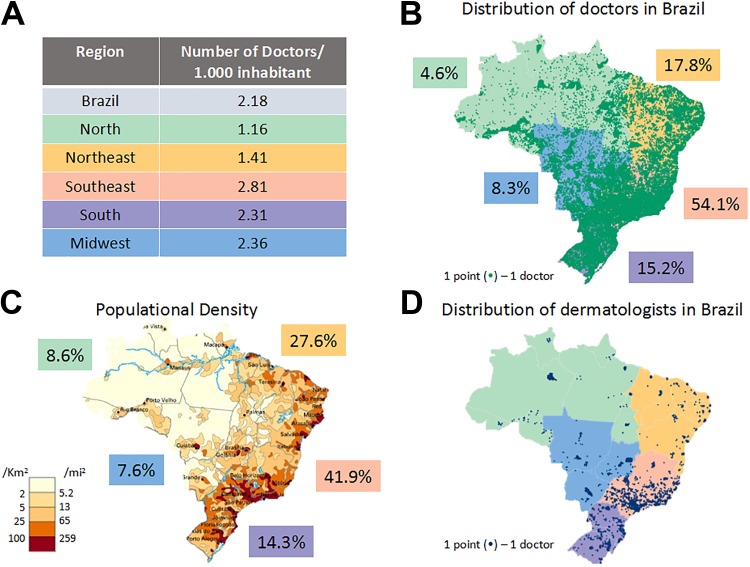
Distribution of medical doctor in Brazil. (A) Number of medical doctor for each 1000 inhabitant separated for region. (B) Distribution of medical doctors in Brazilian territory with the percentage for each region. (C) Population density with the percentage of each region. (D) Distribution of dermatologists in entire Brazilian territory.

Therefore, patient characteristics, country extension, and the high number of people to be treated require geographic distribution of therapeutics possibilities for reach the most part of population. According to a survey published in 2018 by the Department of Preventive Medicine of the Faculty of Medicine of the University of São Paulo (USP) together with the São Paulo State Regional Medical Council and the Federal Medical Council, in the Brazilian territory, there was 2.1 physicians per 1000 inhabitants (data acquired in 2017-2018).^10^ Among the 34 evaluated countries, Brazil has a better rate only compared to Turkey (1.8 physicians/1000 inhabitants). Although the overall physician rate in Brazil is not very different from other countries such as the United States (2.7) and Canada (2.5), there is a high diverse distribution of the health-care human resources and facilities within the Brazilian territory, considering its regions (Figure 1A and B). This scenario represents a huge public health-care problem, especially considering treatment procedures that require complex facilities and highly trained medical professionals.^10^

Among the 451 777 registered physicians in Brazil, 8317 are dermatologists. For a population of 207 660 929 inhabitants, it leads to 4 dermatologists per 100 000 inhabitants. The distribution of the population density is showed in Figure 1C and, for comparison, the distribution of dermatologists is shown in Figure 1B. One can observe several hollow areas, meaning that a huge number of cities (mainly in the country side) and patients have none specialist to detect and treat skin cancer lesions. The lack of specialists to diagnose NMSC lesions means that the estimate number of cases in Brazil is certainly inaccurate. It also means that a patient with a suspicious lesion may have to wait long time to get the correct diagnosis.

The logistic for the patients diagnosed with NMSC to receive treatment may be complex. As we can see from the map, patients from certain locations need to travel long distances to receive adequate health care. It is seen then the importance of seeking effective prevention strategies and new treatment modalities, besides good planning for the allocation of medical resources.

As a great treatment modality, there is photodynamic therapy (PDT), a very promising option for local treatment of tumor lesions. Photodynamic therapy is based on the activation by light of a photosensitive compound leading to the production of reactive oxygen species, which will induce tumor cell death. Cure rates using this technique for the treatment of superficial BCC, using topical medication ranging from 70% to 90%,^11-14^ with improved cosmetic results compared to other procedures.^11,15^ Photodynamic therapy for the treatment of superficial BCC has also been shown as a lower cost technique than surgery since it usually does not need to be undertaken in the operating room and the assistance of qualified personnel, which leads to a reduction in the direct and indirect costs.^15^

Motivated by PDT cost-effectiveness and the worrisome Brazilian health-care situation with NMSC, our group started, in 2011, an initiative to use PDT to treat BCC lesions over the whole national territory, aimed the implementation of new technologies for the screening and treatment of BCC lesions. In addition to PDT, a new equipment using fluorescence was developed and added to treatment system. The fluorescence was used as a form of differentiating normal tissue from abnormal tissue and also to monitor the PDT, using a fluorescence-guided procedure. The population could be benefit greatly with this modern, effective, and lower cost therapeutic modality for the treatment of BCC.

In this article, we present the main results achieved along the 6 years of the so-called “PDT Brazil” multicentric project, comparing the treatment outcome between centers that already had experience with PDT and those that were introduced to the technique through this project. Still, according to the experience of the centers, the response depending on the BCC subtype was also compared. Preliminary results and the initial achievements of the project implementation were addressed in previous publications,^14,16^ and we provide an update on this investigation and the challenges faced by this pioneer study in Brazil. The new developments and arguments for continuation of the project are discussed. We also present a brief discussion on how this type of grant is important for the development of national technology and their dissemination as modalities of cancer treatment in Brazil, the difficulties faced during the project, and why PDT should be incorporated to the unified public Brazilian health system (SUS, from Portuguese, *Sistema Único de Saúde*).

## Material and Methods

### Study Design and Strategy Model

**Figure 2.**
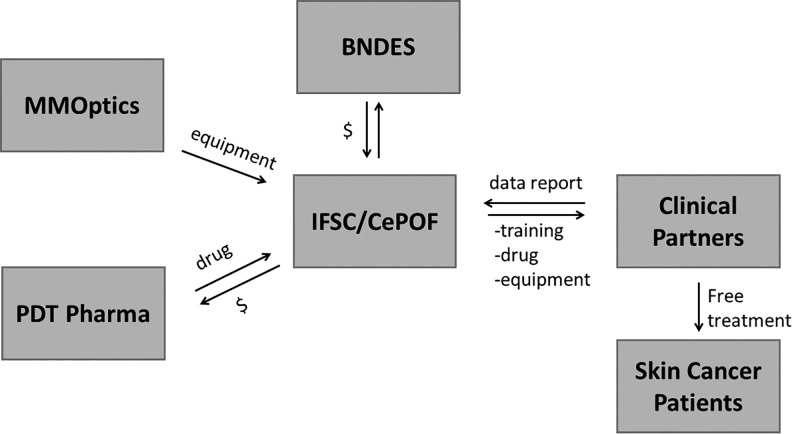
Strategy model including the relationship among university, companies, and clinical partners.

With the objective of stimulating innovation and supporting areas of strategic interest for Brazil, in 2010, the Brazilian Development Bank (BNDES, *Banco Nacional de Desenvolvimento Econômico e Social*) approved the financing to carry out the “PDT Brazil” project. The project was coordinated by the Sao Carlos Institute of Physics at USP, in conjunction with the Skin Department of the Amaral Carvalho Foundation Hospital, and involved researchers, health-care professionals, companies (MMOptics and PDT Pharma), and patients with skin cancer. Figure 2 shows how the relation between the partners was organized.

The first stage of the project was to look for potential clinical partners for the project and to establish a partnership with teams from both public and private health-care institutions across Brazil. Several methods were used for the initial contact: Clinicians and related professionals were contacted during research and clinical meetings, by means of the professional societies of cancer and dermatology. Such contacts were performed in conferences dedicated to those fields and by personal contact between researchers. A compact flyer distributed also helped to disseminate the program.

After this stage, the groups were trained in 2 different approaches: part of the groups was trained at the Center of Photodynamic Therapy in the Skin Department of the Amaral Carvalho Foundation Hospital, and part *in loco*, at the partner facilities. This new group always was formed by a medical doctor who was responsible for the service and reports, and whenever possible, the nursing team also received the training.

A multidisciplinary team formed by at least 1 physicist and 1 health-care professional, both experienced in clinical PDT and its mechanisms, were responsible for training the partner centers. The training course consisted in a theoretical part and a practical part. A 90-minute theoretical course was presented, addressing the fundamentals of PDT and photodiagnosis, pharmacology of aminolevulinic acid (ALA) and methyl aminolevulinate (MAL) as protoporphyrin (PpIX) precursors (an endogenous photosensitizer), clinical protocol, expected and adverse reactions, equipment operation, biosaf ety, and data collection. A full-printed instruction and a literature collection were delivered to all trained partners. After the presentation, the teams had the opportunity to ask questions and discuss. For the clinical hands-on approach, the coordination requested the scheduling of at least 2 patients with lesions fitting the characteristics required by the protocol in order to show all the important steps of the clinical protocol.

Before the beginning of the clinical procedure, our team evaluated if the lesion and patient showed all the inclusion criteria. The patients were oriented concerning the clinical study, the immediate and mediate response, and the potential risks involved, and if in agreement, they signed a written inform consent. The treatment procedure was carried out by the partner team under supervision of the coordination team. The starting cooperative activity including a supervised clinical activity seems fundamental to insure the continuation of the treatment by themselves.

The drug and the equipment used in this project were acquired through collaboration established with a pharmaceutical company specialized in producing PpIX precursors, the PDT Pharma (Cravinhos, Brazil), and an optoelectronic company, specialized in developing medical devices, the MMOptics (São Carlos, Brazil). The chosen PpIX precursor was the MAL 20% in cream (accompanied by a label showing the designation and storage information of the lots inspected and notified by Brazilian Health Surveillance Agency [Agência Nacional de Vigilância Sanitária (ANVISA)]); the equipment used was the LINCE that contains a dual platform, including a light source at 635 nm for the PDT procedure and a fluorescence viewer with a light source at 405 nm; this device has been described elsewhere.^17^ Using the same drug and the same light source was crucial to achieve reliable results in this multicenter project.

To standardize and facilitate the collected data organization of treated patients, a report form was created and presented to the partners during the PDT procedure. They were instructed to fill the form with patient data, lesions localization and characteristics, pain scale during PDT, pictures, follow-up appointments, and other pertinent observations. The partners were instructed to periodically send their progress report and to immediately notify the coordination about any issues concerning doubts, patient unexpected side effects, device technical problems, or any other upcoming problems.

### Patients Selection

Patients were selected by the following criteria: men and women older than 18 years, who were not immunosuppressed, women who are not pregnant or lactating, and presenting superficial (less than 2 cm wide) or nodular (up to 2 mm thick) BCC lesions. After clinical evaluation and histopathology, patients presenting biopsy-confirmed lesions were included in the study and referred to PDT, with clinical follow-up for 6 months. The approval of the Ethics Committee at Fundação Hospital Amaral Carvalho was obtained as required (protocol number 112/11 of 73th Ethics Committee Meeting on October 28, 2011, and protocol 140/11 of December 1, 2011), and the study was conducted in compliance with current Brazilian regulations. Moreover, each center needed to submit the study for their local Ethics Committee to have their own approvals.

### Treatment Procedures

The lesions were prepared before treatment to facilitate cream permeation and light penetration to the target tissue. Each lesion was cleaned following the standard protocol of each center and then debulked with a scalpel blade, previously to MAL cream application. All extension of lesion received a thin layer of cream (about 1 mm thick), and the lesion was occluded with a polyvinyl chloride plastic film, aluminum foil, and bandage to avoid exposure to ambient light.

Three hours after MAL application, excess of cream was removed. The presence of endogenous porphyrins in the tumors was confirmed by visual inspection of the characteristic red fluorescence emission using the blue light excitation of LINCE. The lesions were then exposed to red light for 20 minutes at 125 mW/cm^2^ (150 J/cm^2^). After 7 days, the patients were evaluated and received a second PDT session, following the same procedure of the first session. This second sessions concluded the treatment.

Thirty days after the second session, the treatment response was evaluated. Both clinical and histological evaluation were performed; a 2-mm punch biopsy was taken to evaluate the presence or absence of BCC. The treatment was considered as a “complete response,” where both clinical and histological evaluation showed no residual BCC. In case where the 30-day analysis showed a residual BCC, the response of the treatment was considered “no response.” For the lesion classified as “no response,” the patient was referred to surgery. Patients presenting “complete response” were followed and new evaluation performed after 6 months. Therefore, the data were obtained by classification of the treatment outcomes, regarding the results observed for histopathology from all lesions reported by each center. Each lesion was classified according to its response to the protocol: Full response, partial response, or no response, and the percentage was calculated based on the analysis over the total of lesions reported.

## Results

**Figure 3.**
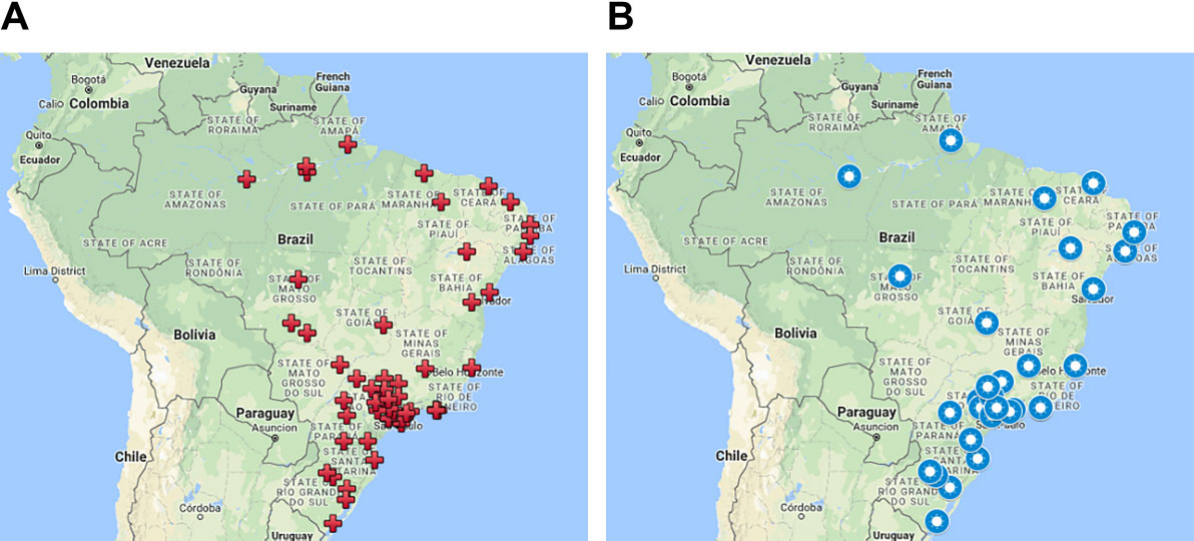
Geographic distribution of partner centers (A) trained for this program and (B) that still active, respectively.

We present the conclusive results collected in 6 years of a multicenter project. A total of 72 centers were trained in different regions of Brazil (North 4, Midwest 6, Northeast 11, South 11, and Southeast 40) and, currently, 42 centers remain active, as shown in Figure 3.

During the execution of this project, a total of 1647 lesions were treated throughout the national territory. Of these, from 2011 to 2015, only in Skin Department of Amaral Carvalho Hospital (a tertiary and oncology hospital), 600 patients were treated according to the standard PDT protocol. Of the 600 patients, 16 could not be able to be reevaluated. The other centers treated the rest of the lesions, with 80.5% of them being BCC treated under the same protocol, including biopsy after 30 days, totaling 843 BCC lesions.

The distribution of the population included in this study was categorized according to gender, age, and skin phototype. Among the BCC lesions treated by our program, 57% were of female patients and 43% male patients.

**Figure 4.**
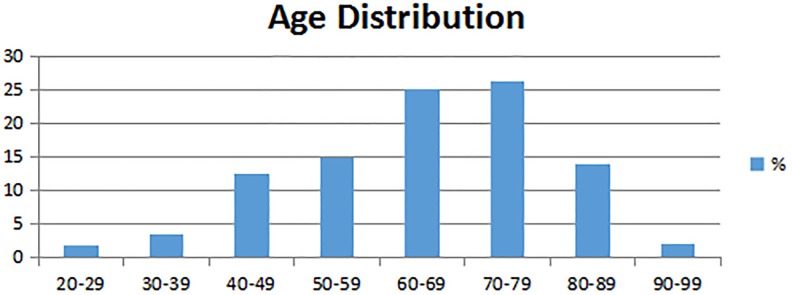
Distribution of the population included in this study, according to age intervals.

Figure 4 shows the age distribution for the population of this study, where 67% of included patients were older than 60 years. This distribution is closely related to the overall incidence of the BCC in Brazil.^2^

Patients were also categorized based on their phototype classification according to the Fitzpatrick scale. In our study, 50% of treated patients were phototype II, 25% were phototype III, 19% were phototype I, and 6% were phototype IV. No patients with phototype V were presented in this study.

**Figure 5.**
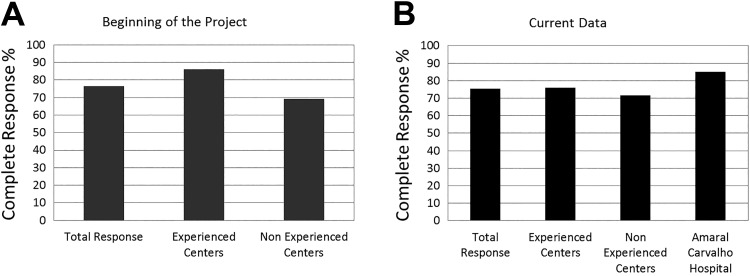
Percentage of complete response for BCC lesions treated with PDT according to the center experience. It is showed the percentage obtained (A) in the beginning of the project, published before^14^ and (B) in the end of the project, with the current data. Amaral Carvalho Hospital is considered only the current data because it is the most experienced center. BCC indicates basal cell carcinoma; PDT, photodynamic therapy.

The reported difference in the treatment response was mainly related to the larger number of adequate lesion indications by experienced centers (ECs), that is, lesions within the protocol: small BCC (with less than 20 mm diameter) and low infiltration (maximum thickness of 2 mm). After 4 years, the difference between EC and non-experienced center (NEC) diminished, as shown in Figure 5: 76% of complete response for ECs and 71.5% for NECs. Therefore, analyzing the 843 lesions treated under the standard protocol for all centers (excepting Amaral Carvalho Hospital), 642 obtained complete response (76.15%) and is showed in Figure 5 as total response for these centers.

Considering the 584 lesions treated by the most EC, Amaral Carvalho Hospital, there was 86% of complete response, with complete follow-up after clinical evaluation and the 30-day biopsy. This result means that a residual BCC was observed in only 80 lesions, which were considered “no response.” This result is also shown in Figure 5.

**Figure 6.**
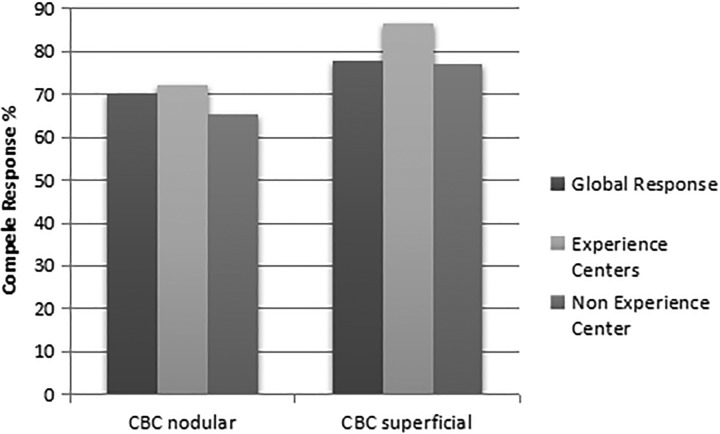
Percentage of complete response for BCC superficial and nodular lesions according to the center experience. BCC indicates basal cell carcinoma.

Despite superficial BCC being the most suitable skin cancer lesions to be treated with PDT, nodular BCC lesions also present excellent outcome. The evaluation of these type of lesions treated with the referred clinical protocol was made for all centers except Amaral Carvalho Hospital, therefore totaling 843 lesions. Figure 6 shows that superficial lesions, in general, had higher percentage of complete response than nodular lesions (77.8% for superficial lesions in contrast to 70% for nodular lesions). Nodular lesions are generally thicker than superficial ones and that may explain this difference. Consequently, the percentage of complete response was higher for superficial independently of the center experience; nodular lesions had complete response rates of 72% for ECs and 65% for NECs, while for superficial lesions, ECs had complete response in 86% and NECs, 77%.

Regarding the body location of BCC treated in this study, 78% of lesions were found in the head and neck region, 13% in chest and back regions, and 9% in limbs.

**Figure 7.**
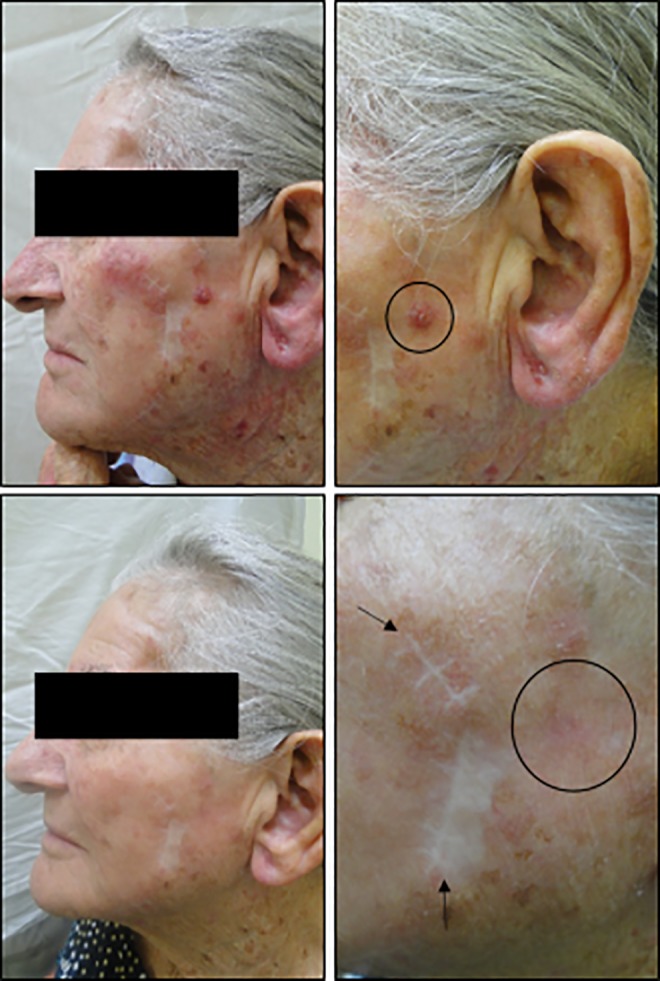
L.M.P.M, female, 75 years old, nodular BCC lesion in auricular region, before and 30 days after the PDT procedure. The circle shows the region that was treated and the arrows indicate previous surgery that the patient had to remove others BCC lesions. BCC indicates basal cell carcinoma; PDT, photodynamic therapy.

Figure 7 shows the comparison between aesthetics results of surgery and PDT to treat BCC lesions in a 75-year-old patient. On top of Figure 7, the area before treatment is shown and bellow is the same area after 30 days. It is highlighted the scar in a previous surgery and the good healing process of PDT, with completely recovered skin.

**Figure 8.**
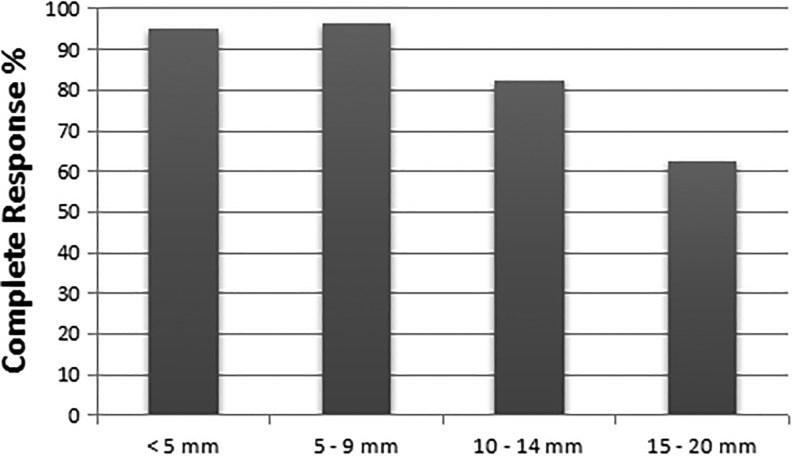
Percentage of complete response according to the lesion size after 6 months of the treatment.

Our results indicate that, as time goes by after treatment, patient tracking becomes a challenge and the real number of treated patients could be much larger than reported by the centers. As several patients do not return for the 6 months evaluation. Considering the reports of all centers (excepting our reference center, Amaral Carvalho Hospital), the results of biopsies collected 6 months after PDT totalized only 73 lesions and they are shown in Figure 8 with their respective sizes. It is clear that the size of lesion influences directly the total response of PDT, where larger lesions (15-20 mm) had showed a lower response rate.

Although the protocol of the project was restricted to cases of BCC up to 20 mm in diameter in order to obtain satisfactory results, we could identify also premalignant and BCC lesions larger than 20 mm, which were included in this study. Two hundred and four lesions identified as Bowen disease, actinic keratosis, pigmented BCC, among others were reported during the time span of this study. Among these lesions, 88 had biopsy collected 30 days after PDT and 29 were only clinically assessed; among them, 82 presented complete response, leading to 70% of successful treatment of lesions different from the protocol preestablished for this study, always in NMSC.

## Discussion

With almost 20 years of experience in PDT to treat malignant lesions, our group has cumulated the knowledge on the effectiveness of this technique to treat NMSC and its advantages, such as being less costly and easier to apply than surgery. The initiative of the “PDT Brazil” project was, then, to implement several PDT centers all around the Brazilian territory to treat BCC lesions, providing equipment, drugs, and training course, leading to a more standardized PDT procedure by following the established protocol.

Among these 72 initial centers, only 22 were universities or research centers, which meant that the majority of our partners were not involved with any type of academic work. However, after participating in the project, several reports showed the involvement of members with some academic project, which was considered a great secondary effect of the PDT Brazil project implementation: the interest for research after this contact with the technique.

The first results of the PDT Brazil project, published in 2014,^14^ compared the treatment outcome regarding the previous experience of the centers and showed that, at that point, ECs reached 86% of complete response, while NECs reached 69.2%. Even with all advantages offered by PDT, during the course of our project, the general lack of knowledge by the medical community in Brazil concerning the technical details of PDT was a remarkable fact. The responsible physician of each center, at the moment of the application to take part in the project, assigned him- or herself and his or her local team as an EC or NEC regarding their own previous knowledge and/or practice with PDT for at least 1 year. Among the 72 centers trained, only 54% of them declared previous experience, which reflects that lack of knowledge, probably because topical PDT approaches are relatively new in dermatology, especially in Brazil.

The results of the present study show an impressive increase in the number of patients, reached since the partial results published in 2014. The difference between the ECs and NECs since then decreased; however, the factor continued to influence in almost 10% of BCCs response. The results suggest that ECs are more judicious to select lesions to PDT.

If we analyze the cure rate for surgical excision, the standard treatment for BCC, it is about 96%,^7^ while in our study with this present protocol, PDT presented about 80.18% of complete response considering the 1647 treated lesions. In spite of this, if we consider the long waiting times for the surgery that may lead to complications for the patients and a further increase in the costs involved, PDT can be considered an advantageous therapy. Especially for Brazilian health-care services reality, since SUS usually has a long waiting time for surgeries due to lack of resources, the complete treatment of 80% of cases with this simple procedure may help to reach treatment for 100% of the cases, which today is not possible.

Due to the high incidence of NMSC, with more than a hundred thousand new cases every year only in Brazil,^2^ it is impossible for SUS to handle this demand efficiently only with the application of surgical procedures. As reported by de Souza et al in 2011,^18^ an estimate of the costs for the treatment of NMSC in São Paulo state showed that 95% of the lesions are diagnosed in early stages and, in spite of this, due to the high number of patients, it represents a heavy financial burden of about BRL$ 37 million (about USD$ 20 million at that time) to the public health-care system per year. Currently (2018), this cost is estimated to reach over BRL$100 million per year.

An important issue is that among the 451 777 registered physicians in Brazil, there are 8317 dermatologists and they are distributed in big centers, as showed in Figure 1. This means a waiting for BCC treatment with surgery of more than 3 months, as reported by 2 references center in São Paulo state, Amaral Carvalho Hospital, in Jaú, and Hospital of Cancer, in Barretos.

In this scenario, the importance of the development and implementation of new treatment modalities with lower costs becomes essential, and PDT shows up as a very promising alternative since it is an outpatient clinic procedure that involves less material, equipment, and, mainly, human resources. Photodynamic therapy may reduce the costs in more than 50% of surgeries,^19^ creating possibilities to decrease the waiting time and spreading assistance over a large geographic area, and more important, it may reach 80% more people with small lesions.

Another recognized advantage of PDT over surgery is the better aesthetic outcome, with an excellent healing process, particularly for the treatment of primary lesions. This, in fact, is a unanimous opinion among physicians who have performed PDT as well as surgery.^11,20^ Generally, BCC is not an aggressive lesion, with low mortality rates; however, the surgical treatment may result in physical deformity if the lesion is not treated while in early stages, resulting in extensive morbidity that includes reduction of anatomical functionalities.^21^ The aforementioned advantages of PDT are decisive to the treatment acceptance by patients.

**Figure 9.**
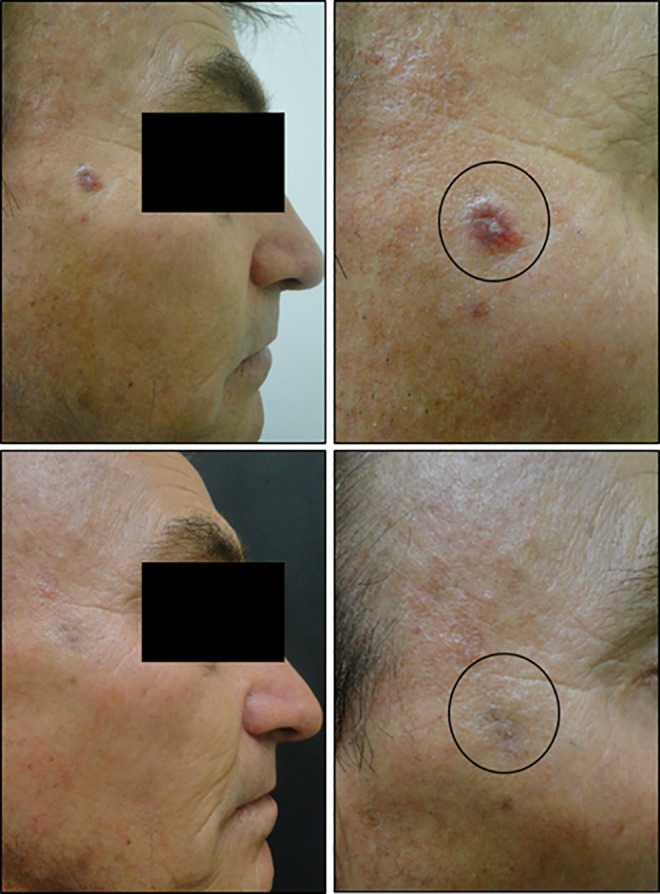
A.C.S., male, 49 years old, nodular BCC lesion in zygomatic region, before and 30 days after the PDT procedure. The circle shows the region that was treated. BCC indicates basal cell carcinoma; PDT, photodynamic therapy.

The high incidence of BCC in the head and neck is associated primarily with sunlight exposure. Surgical procedure on these areas often results in a significant morbidity as it may compromise facial functionality and aesthetics. Whereas skin has great importance from the psychological point of view, influencing the emotional life of patients, psychic disorders may happen after the occurrence of deformities caused by cancer on those sites, which highlights how important is to offer treatments with good aesthetics results in those cases. Figure 9 shows a patient presenting facial BCC lesions and the excellent aesthetic result with complete elimination of lesion after the PDT treatment.

The surgery of cutaneous facial reconstruction of BCC lesions needs to be performed by a well-trained specialist, while PDT is an easy-to-perform technique, requiring only a simple training, mainly to deal with the equipment—and results are exceptionally improved with time, as the medical doctor becomes more experienced, as our study shows. In addition, the aesthetic outcome of the surgery, depending on lesion extension, depth, and location of occurrence may also be a negative factor. In such a case, an alternative is to perform PDT over multiple sessions for the reduction of the lesion, enabling to subject the patient to a less invasive surgical procedure.^22^

**Figure 10.**
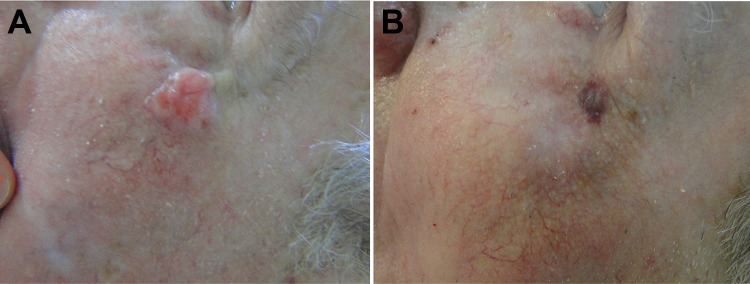
A 72-year-old patient with Alzheimer who could not undergo surgery and was submitted to PDT. (A) Before treatment. (B) After 15 sessions. PDT indicates photodynamic therapy.

Photodynamic therapy could also be performed in patients with several debilities, which makes it impossible for him or her to undergo surgery. In cases where there are family doctors and the physician goes to the patient’s house, PDT is a possibility to offer a treatment at home. In a letter published in 2014,^23^ we presented a special case when PDT was chosen due to the absence of other possible therapeutical approaches and to its advantages as a topical application, possibility of repeated sessions, and few adverse reactions, enabling the application at outpatient clinic facilities. After 15 PDT sessions with the same protocol used in the PDT Brazil project, it was observed a full lesion reduction, with excellent cosmetic outcomes, suggesting the possibility to apply multiple PDT sessions for the treatment of these lesions, especially in cases for which other therapies are contraindicated. Among the treated patients, there is one clear example, in which the treatment response of a 72-year-old patient with Alzheimer disease for 10 years and with sclerodermiform is shown in Figure 10. This technique is not usually indicated for this subtype treatment due to the more aggressive behavior. However, PDT could be performed in cases where there is no possibility of surgery, showing one more advantage compared to surgery.

An interesting finding refers to the gender of patients treated. When looking at the population of patients covered by the project, one can see that 14% more female patients were treated. The larger number of female patients can be related to the fact that women tend to be more concerned about the development of health issues, particularly skin cancer lesion, and usually seek and carry out treatment more frequently than men, which results in an earlier diagnosis.^24,25^

Besides the financial challenges concerning this particular project, several other difficulties are faced when a multicenter clinical trial is conducted. The diversity of infrastructure available in the different centers is a notorious fact. The project members visited several localities spread in the Brazilian territory and they found facilities with different degrees of resources due to different economic situations over the country. These differences hamper the project data collection, as it would be necessary local cost matching for, for example, performing periodic biopsies (before PDT, 30 days and 6 months after PDT) in order to evaluate the treatment response. It is not rare in Brazil to find cities in which there are no clinical laboratories able to perform this type of examinations and, when that is the case, the material collected in the biopsy has to be sent away for long distances for examination, and the waiting times to obtain the results is impracticable for clinical routine. Where histopathology examinations were impractical, the recommendation for centers, in order to avoid these barriers, was to perform only clinical analysis by the specialist physician (dermatologist or oncologist) before the lesion treatment, leaving biopsies for response evaluation only. This reality is also present in most of Latin America countries. Therefore, all observations for Brazilian territory and population match realities met beyond Brazilian borders.

Other reasons might be suggested to explain the decrease in the number of biopsies 6 months after PDT. It is a common observation by physicians that, when patients notice a good treatment response of the lesion, some patients do not return for the follow-up, as they believe to be cured—which is a particular issue among low education patients, unaware of the risks of such behavior. In addition, there are cases where patients are unable to return for the follow-up due to financial/mobility issues or even to death related to other diseases or natural causes, mainly because the majority of these patients are elderly individuals, as presented in Figure 4.

Another reason for this low number of lesions with long-term biopsies could be the lack of this information in the reports provided by each center. As the project involved more than 70 centers, we believe that part of them just not provided all the data the coordination requested.

This project also encouraged developing countries in South America, such as Bolivia, Chile, Ecuador, El Salvador, Colombia, Cuba, Mexico, Peru, and Venezuela, to take PDT as a clinical approach to places distant from health-care facilities in population centers, as well as to allow greater interaction between physicians and universities.^16^ This study allowed the interaction of our research group, responsible for the project coordination, with medical doctors, especially dermatologists, who were interested in getting started with PDT. Today, PDT became a well-recognized technique in Brazilian dermatological communities and even referred as treatment option.

The number of centers involved, and therefore, of lesions treated, was expected to be larger. However, the implementation of a single national platform for registration of research clinical projects and the consequent requirements of local adaptation by ethics boards and institutional procedures were a challenge for several groups to be included in the study. It is not the scope of this study to discuss this aspect further as it was a punctual fact, but such a remark may help highlighting the role that documental aspects may represent in arranging large multicenter studies.^26^

In this meanwhile, a number of initially committed partners ended up giving up or got delayed by the adaptation between the former and the new submission processes, creating repeatedly refusal of submissions by local research ethics committees (RECs). Without this approval by the RECs, the researchers would not be able to start the study, which ended up in several partners withdrawing their collaboration. Nevertheless, the centers that managed to have the approvals engaged the project enthusiastically, moved by the chance to contribute to the fostering of PDT, and by the clinical results obtained, and their reported results enabled this initiative to start the process to make PDT available on SUS.

The present multicenter clinical study involved a high diversity of medical team expertise on PDT but also most of them were performing clinical research for the first time. This scenario resulted on some limitations for the project workflow and final analysis. A higher number of lesions were treated, but due to the uncompleted medical reports, these data could not be included. Main missing data were related to the lack of the histology report at the 30th day after the second PDT session and delayed or missing follow-up session. Other relevant limitation was the indication of borderline BCC lesions, mostly performed by the NECs at the beginning of the study. In this case, when we identified an uncorrected patient selection, our clinical team worked remotely with the medical doctor to once again discuss the inclusion criteria and closely evaluated further patient enrollment. In the end, these limitations did not globally affect the study execution, and the overall results and analyses are here presented.

### Essential Partnerships

The project also promoted 2 PDT-related Brazilian products: the medication and the equipment. PDT Pharma, a pharmaceutical company specialized in photosensitizers and PpIX precursors, emerged as pioneer in the field in Brazil. The company is producing ALA and MAL, the 2 main PpIX precursors for PDT. The approval of these medications at ANVISA is directly related to the progress of the project here reported. Further, LINCE was developed for this project by MM Optics, aiming to facilitate spreading PDT among the medical doctors by making the use of a fluorescence viewer to evaluate skin lesions and also to monitor PDT available concomitantly to the treatment device—which is also the first device to do so worldwide.

Certainly, the success of the project is owed in part to the collaboration with a company with experience in the field and a history of innovation, besides the support for the creation of PDT Pharma. This made the project very well succeeded from both clinical and commercial points of view, which made possible the development of national technology and the dissemination of PDT. The experience of our groups and the involvement of a university in the development of partnerships showed the important role of research in universities for the society and, undoubtedly, played an important role for the BNDES approval of the financial support of the project.

In this sense, despite the fact that the main goal of this project was to train physicians and health-care teams for the use of PDT for skin cancer treatment by a standardized clinical protocol, this project went further and contributed decisively to push forward the approval of the first national medication for PDT in Brazil, in addition to foster the Brazilian industry in this field.

### Final Remarks

*Sistema Único de Saúde* is one of the largest public health-care systems in the world. It covers from simple outpatient care to organ transplantation, ensuring universal and free access to the entire population of the country. It was created in 1988 by the Brazilian Federal Constitution, based on the welfare state idea, and can be considered one of the greatest social achievements of the Brazilian.^27^

Actual universality is still an ideal to be achieved. In order for SUS to become really universal, it is necessary to further the process of extending coverage of services so that they become accessible to the entire population. Such an endeavor demands to eliminate economic, cultural, and social barriers that are interposed between the population and services. From the economic point of view, although the service is provided by government, a large part of the population is poor, living in small localities with low economic development, or inhabits the periphery of large cities and does not have minimum conditions to access services, for reasons such as the inability to afford by themselves the transportation necessary to reach a health-care unit. From the social–cultural point of view, the main barrier is the communication, as a relevant part of the population has no educational and cultural conditions that enable the dialogue with health-care professionals, which in turn implies the difficulty of understanding basic procedure to be adopted for risks prevention and health recovery.^28^

The great cultural heterogeneity and demographic, socioeconomic, and political interferences in Brazil can limit the quality of care provided by SUS, evidencing problems such as exhaustion of the health-care system, which result in long hospital queuing for medical appointment, with expressive worsening of patients’ clinical status.

Investments have been made on SUS, as it is in permanent expansion in order to reach a larger part of the population. In the meantime, it has an ever-increasing demand of patients, which affects the quality of the service and the waiting times for the patients to access certain types of treatment or examinations, such as biopsies in order to obtain an accurate diagnosis of cancer lesion type. When patients are finally diagnosed, if the required procedure demands hospitalization, patients need to wait again for a vacancy in order to undergo a procedure. Thus, treating an NMSC may take a very long waiting time for the patient and consume extensive resources. Therefore, considering this scenario, the development of new technologies that demand less financial resources and operational time can represent an enormous contribution to the Brazilian health-care system, with undeniable improvement of population welfare.

The promising results obtained in this multicenter study motivate us to continue work on new protocols to improve PDT complete response rates. A pilot study with single visit resulted in a higher complete response rate and is in preparation for publication. With the acquired experience by the many professionals involved in the localities, new protocols shall be easier adopted, resulting in considerable improvement. The final result using PDT in BCC of small extension with national technology shall get close to the results by the gold-standard surgical procedure. This may be a great encouragement for the inclusion of PDT as a main choice in Brazil.
